# Changes in microbial community during hydrolyzed sludge reduction

**DOI:** 10.3389/fmicb.2023.1239218

**Published:** 2023-09-01

**Authors:** Shaomin Liu, Jiating Wu, Ziyan Hu, Mengyu Jiang

**Affiliations:** ^1^School of Earth and Environment, Anhui University of Science and Technology, Huainan, China; ^2^State Key Laboratory of Mining Response and Disaster Prevention and Control in Deep Coal Mines (Anhui University of Science and Technology), Huainan, China

**Keywords:** hydrolysis, sludge reduction, microorganisms, community structure, diversity

## Abstract

In this study, the effects of different enzymes (lysozyme, α-amylase and neutral protease) on sludge hydrolysis efficiency and microbial community in sequencing batch reactor (SBR) were introduced. The results showed that the hydrolysis efficiencies of the three enzymes were 48.5, 22.5 and 31%, respectively, compared with the accumulated sludge discharge of the blank control group. However, it has varying degrees of impact on the effluent quality, and the denitrification and phosphorus removal effect of the system deteriorates. The lysozyme that achieves the optimal sludge hydrolysis effect of 48.5% has the greatest impact on the chemical oxygen demand (COD), total nitrogen (TN), and nitrate nitrogen (NO_3_^−^-N) of the effluent. The sludge samples of the control group and the groups supplemented with different enzyme preparations were subjected to high-throughput sequencing. It was found that the number of OTUs (Operational Taxonomic Units) of the samples was lysozyme > α-amylase > blank control > neutral protease. Moreover, the abundance grade curve of the sludge samples supplemented with lysozyme and α-amylase was smoother, and the community richness and diversity were improved by lysozyme and α-amylase. The species diversity of the sludge supplemented with lysozyme and neutral protease was great, and the community succession was obvious. The introduction of enzymes did not change the main microbial communities of the sludge, which were mainly Proteobacteria, Actinobacteria and Bacteroidetes. The effects of three enzyme preparations on sludge reduction and microbial diversity during pilot operation were analyzed, the gap in microbial research was filled, which provided theoretical value for the practical operation of enzymatic sludge reduction.

## Graphical abstract

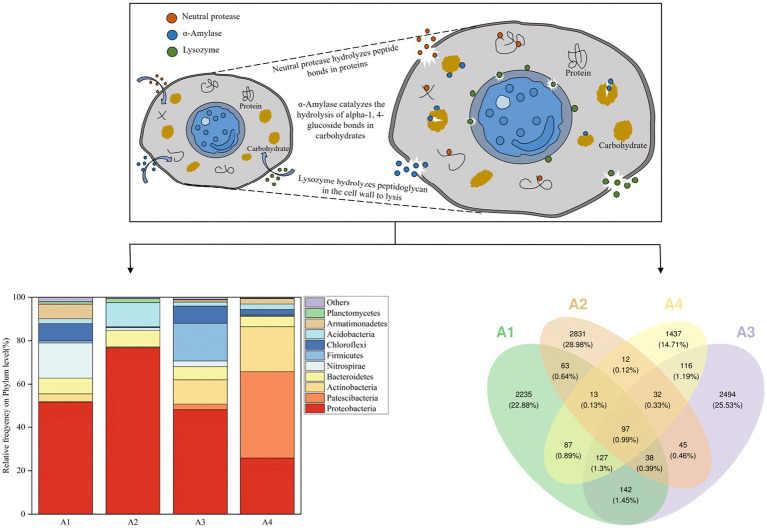

Effects of enzymatic hydrolysis on microbial community structure.

## 1. Introduction

Residual sludge is the main by-product of wastewater treatment plants, which originates from the biomass growth caused by the degradation and consumption of organic pollutants in wastewater. In the activated sludge process, the ratio of COD to sludge is about 0.3–1.2 kg TSS/kg COD, and the sludge production is huge, which poses a direct or potentially significant threat to the ecological environment (Zou et al., 2022; Apollo et al., 2023). As the main tank of domestic and industrial wastewater, sewage treatment plants contain a large number of polluting substances and are enriched into activated sludge, making these sludge compositions complicated without proper treatment and disposal (Huang et al., 2023; Wang et al., 2023). It not only contains high water content, sediment, and plant and animal residues, but also a large number of microorganisms, pathogens, heavy metals, and other toxic and harmful substances. Special components resulting in the residual sludge has long-term toxicity and non-degradability, so it must be treated and disposed of in an effective and safe way (Yu et al., 2023). In 2022, sludge production has exceeded 65 million and has been growing in China (Pang et al., 2021), but for a long time, the research on sludge emission reduction has lagged far behind that of developed countries. The commonly used sludge end treatment and disposal methods require high operating costs and land use (Quan et al., 2022). Therefore, the sludge *in-situ* reduction technology has been widely used as a way to reduce excess sludge from the source (Liu Q. et al., 2022; Liu X. et al., 2022; Liu Z. et al., 2022). It not only reduces the environmental impact and economic burden of the sludge treatment process, but also does not require extensive modification of the original process (Jiang et al., 2021).

In sludge digestion, hydrolysis is considered to be the primary rate-limiting step in sludge lysis pretreatment, and a large number of studies have adopted the pretreatment method that decomposes sludge and releases organic matter into the aqueous sludge phase (Peng et al., 2018). In the process of sludge reduction, it can regulate cell lysis, hydrolysis, nutrient extraction, sludge improvement and other functions, and simultaneously achieve pollutant removal and sludge reduction (Zhou et al., 2023). Adding uncoupling agents to hydrolysis methods is difficult to degrade, most of them are toxic and harmful substances, ozone is costly, and has limitations in application. Other methods also have inevitable disadvantages. In contrast, enzyme treatment has been extensively studied as a mild, environmentally friendly, and efficient treatment method (Liu et al., 2019). Some researchers found that amylase, neutral protease and other enzymes in different conditions could improve the hydrolysis efficiency of residual sludge and specific types of enzymes catalyze only one defined substrate (Yu et al., 2013; Chen et al., 2015; Lu et al., 2022; Kang et al., 2023a,b). Liu et al. (2019) as well as Song et al. (2013) investigated the catalytic performance of lysozyme on residual sludge hydrolysis and decomposition, and found that it could serve as a lysis agent to effectively reduce the amount of residual sludge produced in SBR systems. In addition, extracellular polymeric substances (EPS), the main component of sludge floc, is easier to extract after enzyme treatment, and the floc structure is looser, which is the key to the reduction process (Kang et al., 2023a,b). These studies proved the necessity and effectiveness of the application of enzymes in sludge reduction and the rationality of the selection of neutral protease, α-amylase and lysozyme in this study. However, most current studies focus on exploring the effects of different enzyme preparations on sludge reduction and dehydration (Pang et al., 2022), ignoring the effects of the addition of enzyme preparations on water quality and microbial community structure. Therefore, this study will use enzyme preparations as a starting point and put them into a pilot experimental device simulating an actual sewage treatment plant. The analysis of effluent quality as well as changes in microbial community structure in the sludge provides new insights for the formation of a more integrated and comprehensive system for enzyme treatment to provide a disposal strategy in future practical wastewater treatment plants operation.

## 2. Materials and methods

### 2.1. Experimental setup

After fresh sludge was taken back to the laboratory, it was cultured for 24 h, then moved into the SBR for incubation. The operation cycle is 8 h, and the operation mode is intermittent inlet and outlet, including 0.5 h inlet stage, 1.5 h anaerobic stirring, 3 h aeration stirring (DO is about 2.0 mg/L), 1.5 h static sedimentation, and 0.5 h outlet. The next cycle starts after the system is static and the water is changed. Three cycles were run daily with regular quantitative mud discharge, and the domestication time was 21 days. In order to ensure the stability and accuracy of water, the experiments were conducted by artificially preparing simulated domestic wastewater with 510 mg/L CH_3_COONa, 82 mg/L NH_4_Cl, 53 mg/L KH_2_PO_4_, and 16 mg/L K_2_HPO_4_ to provide carbon, nitrogen, and phosphorus sources required for microbial life, and added NaHCO_3_ to control the pH of the water in 7.5 ~ 8.5. In addition, appropriate amounts of trace elements were added to the synthetic wastewater, including 44 mg/L MgSO_4_, 100 mg/L FeCl_3_·6H_2_O, 0.12 mg/L MnCl_2_·H_2_O, 0.15 mg/L CoCl_2_·6H_2_O, 0.15 mg/L H_3_BO_3_, 0.12 mg/L ZnSO_4_·7H_2_O, 0.06 mg/L Na_2_MoO_4_·2H_2_O, and 0.03 mg/L KI (He et al., 2021).

### 2.2. Sludge and enzymes

The residual sludge was obtained from Panji Wastewater Treatment Plant, Huainan, China. The sludge characteristics are as follows: pH is about 8, mixed liquor suspended solids (MLSS) is 2,571 mg/L, mixed liquor volatile suspended solids (MLVSS) is 1831 mg/L, COD is 31 mg/L, ammonia nitrogen (NH_4_^+^-N) is 0.657 mg/L, and sludge retention time (SRT) is about 15 d. Neutral protease was purchased from Hefei Bomei Biotechnology Co. Ltd., with an activity of 200 u/mg, the optimum pH of 7.0, and a reaction temperature of 50°C. α-Amylase was purchased from Hefei BASF Biotechnology Co. Ltd., with an activity of about 10,000 u/g, the optimum pH of 7.0 and the reaction temperature of 55°C. Lysozyme was purchased from Shanghai IKA Biotechnology Co. Ltd., with an activity of 33,000 u/mg, the optimum pH of 7.0, and a reaction temperature of 35°C (Kang et al., 2023a,b; Zou et al., 2023).

### 2.3. Experimental method

The purpose of the experiments is to investigate the changes in the microbial community structure of the sludge in each reactor at the end of the reaction cycle run when different enzymes were used to treat sludge in the plant. Four identical SBR reactors with a total reaction volume of 4 L (effective volume of 2.5 L) were used in the experiment. They were labeled as A1, A2, A3, and A4, and the first one was used as a blank control. Then, different enzymes of the same concentration were added to A2, A3, and A4 reactors, respectively. The A2 reactor had 0.2 g lysozyme/g SS, the A3 reactor had 0.2 g α-amylase/g SS, and the A4 reactor had 0.2 g neutral protease/g SS. Before stopping aeration in each reaction cycle, 10% of the sludge mixture was taken out from the enzyme reaction tank, left to settle, and then the supernatant was removed. Then the enzyme reagent was added to the settled sludge, and adjusted to the optimal pH, respectively. After stirring at different temperatures for 30 min in the water bath, the sludge was added back to the drained SBR reactor. The sludge samples were taken from the last day of continuous operation for 24 days for microbiological assays. Each experiment was performed in duplicate and repeated to determine the accuracy of the results obtained.

### 2.4. Analysis methods

#### 2.4.1. Analysis of sludge and water quality indicators

MLSS were determined according to the standard method (APHA, 2005) to reflect the sludge discharge. The sludge volume index (SVI) measured by sedimentation method reflects the sedimentation property of sludge. Water quality indicators, including chemical oxygen demand (COD), ammonia nitrogen (NH_4_^+^-N), total phosphorus (TP) and total nitrogen (TN) parameters, were determined by potassium dichromate method, nano reagent spectrophotometry, molybdate spectrophotometry and potassium persulfate oxidation-ultraviolet spectrophotometry. Nitrous nitrogen (NO_2_^−^-N) and nitrate nitrogen (NO_3_^−^-N) which reflected the nitrification and denitrification ability of the system were determined by the N-(1-naphthyl)-ethylenediamine spectrophotometry and UV spectrophotometry. The pH and DO values were determined by an FE28 pH meter and a JPB-607A portable dissolved oxygen meter, respectively.

#### 2.4.2. Analysis of microbial community diversity

The bacterial composition of the bioreactor was examined by high-throughput sequencing of a 16S rRNA gene library generated by amplification primers for the V3-V4 region of the bacterial 16SrRNA gene. The V3-V4 region of the 16S rRNA gene was amplified with forward primer 338F (5′-ACTCCTACGGGAGGCAGCA-3′) and reverse primer 806R (5′ -GGACTACHVGGGTWTCTAAT-3′). The V3 and V4 regions were chosen because they were reported to represent reliable regions of the full-length 16SrRNA gene in many studies (Plotnikov et al., 2019).

Sludge samples run for 24 d in four devices, A1, A2, A3, and A4, were pretreated to dissolve the DNA, SDS was added to remove impurities and destroy the cells before the cell was broken, and the DNA was split by mechanical crushing method. Meanwhile, Nanodrop was used to quantify the DNA, and the DNA extraction quality was detected by 1.2% agar-gel electrophoresis. Then, specific gene fragments were amplified by polymerase chain reaction (PCR), and magnetic beads were added to the amplified products for purification and recovery. The recovered products were quantified by PCR amplification. The fluorescence reagent was Quant-iT PicoGreen dsDNA Assay Kit, and the quantitative instrument was Microplate reader (BioTek, FLx800). Based on the quantitative results, sequencing libraries were prepared using Illumina’s TruSeq Nano DNA LT Library Prep Kit as required. Finally, high-throughput sequencing was carried out on the computer. This project conducted double-ended sequencing of community DNA fragments, adopted DADA2 and Vsearch for sequence denoising or clustering, used Greengenes database to annotate species taxonomy, completed bioinformatics analysis, and then performed species composition analysis. The specific results of each sample at different species classification levels were obtained. The differences and significance of microbial diversity can be seen from ASV/OTU, species classification and other aspects.

## 3. Results and discussion

### 3.1. Mechanism of action of enzyme preparations

**Figure 1:**
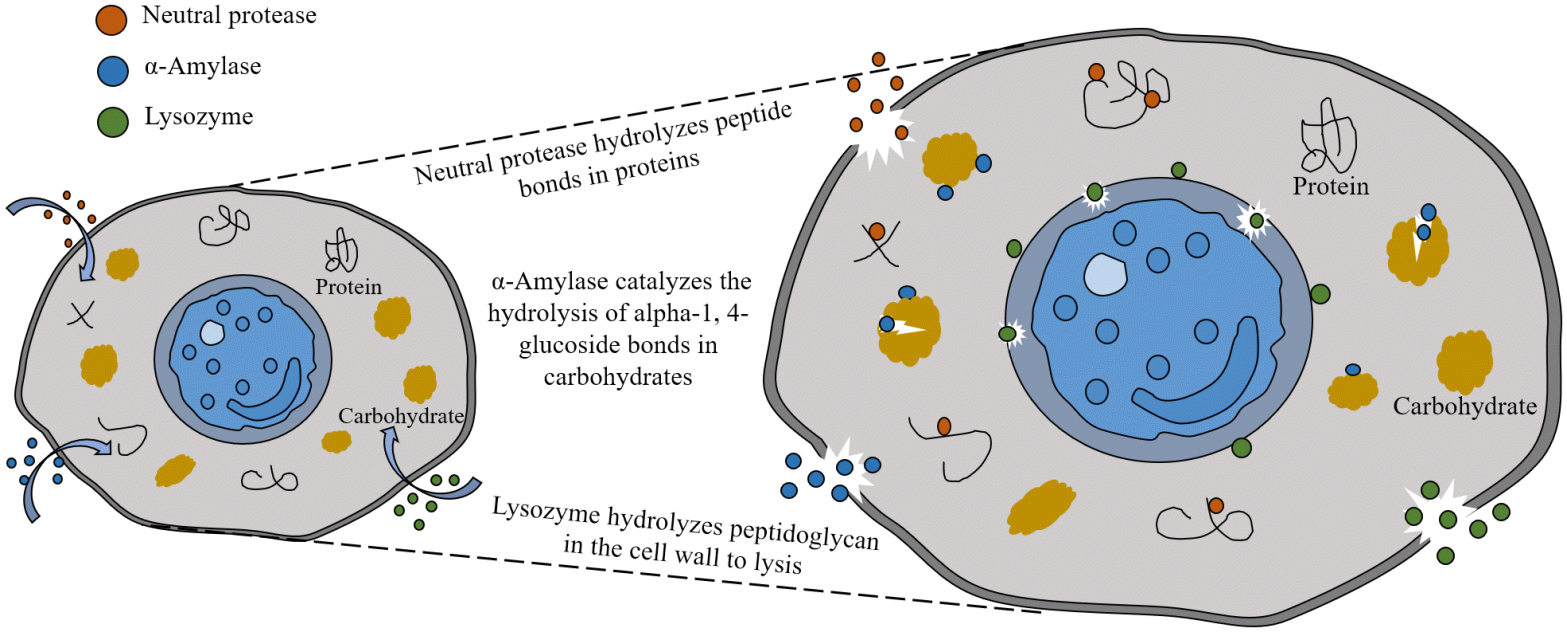
Mechanism of sludge hydrolysis by lysozyme, α-amylase, and neutral protease.

Neutral protease can dissolve. Under certain conditions, using the characteristics of the enzymatic reaction, it can hydrolyze the peptide bonds in macromolecular proteins in sludge, and release amino acids or small molecule peptides (Zou et al., 2023), The catalytic reaction is fast and destroys the stability of the sludge floc structure. α-Amylase, also known as 1,4-α-D-glucan glucan hydrolase, can catalyze the hydrolysis of α-1,4-glycosidic bonds (Plaza-Vinuesa et al., 2019). As a result, the hydrolysis of carbohydrates in the sludge is combined during the reaction process, resulting in sludge reduction. Lysozyme, as known as N-acetylmurastin hydrolase, continuously catalyzes the peptidoglycan hydrolysis of the cell wall by cutting the β-1,4 glycosidic bond, and then dissolves the bacterial cell wall until the cell wall is completely disintegrated. This leads to the release of intracellular substances, and the lysis is completed to achieve the effect of sludge reduction (Shi et al., 2022; Figure 1).

### 3.2. Effects of sludge reduction and wastewater treatment performance

During the experiment, the sludge samples were extracted and measured at regular intervals. The results are shown in Table 1. During the operation, the cumulative discharge of residual sludge in A1, A2, A3, and A4 was 2,668 mg, 1,373 mg, 2067 mg, and 1840 mg, respectively. The reduction of residual sludge in A2, A3, and A4 reached 48.5, 22.5, and 31%, respectively, which proved that adding enzyme preparation could enhance the residual sludge hydrolysis and promote sludge reduction. The SVI values can reflect the looseness, coalescence, and sedimentation performance of activated sludge (Li et al., 2016). When the SVI value is higher than 150 mL/g, expansion will be considered (Han et al., 2018). At the end of the operation cycles, SVI values were 98 mL/g, 72 mL/g, 270 mL/g, and 87 mL/g of A1, A2, A3, and A4, respectively. The sludge particles in the A2 device were dense and inorganic. The sludge in A3 had a pronounced tendency to swell, which may be caused by the filamentous bacteria on the surface, which will limit the operation of the SBR unit. The sediment produced by A4 with the addition of neutral protease had good settling performance.

**Table 1:** Sludge and water quality indicators.

Indicators	A1	A2	A3	A4
Cumulative residual sludge discharge (mg)	2,668	1,373	2067	1840
Final SVI (ml/g)	98	72	270	87
COD of effluent (mg/L)	14.97	41.52	27.12	27.12
TP of effluent (mg/L)	5.38	5.86	9.13	9.34
TN of effluent (mg/L)	14.92	45.69	14.75	7.40
NH_4_^+^-N of effluent (mg/L)	0.60	1.15	0.56	0.73
NO_2_^−^-N of effluent (mg/L)	0.47	0.48	0.53	0.47
NO_3_^−^-N of effluent (mg/L)	14.04	41.95	14.93	9.07

After adding the enzyme preparation, the effluent COD of the A2, A3, and A4 enzyme reaction tanks was higher than that of the A1 reaction tank, mainly because the sludge was hydrolyzed and the microbial cells were cleaved and died. As a result, intracellular organic matter was released, leading to an increase in COD. On the one hand, the increase in TP content was due to the reduction of microbial biomass and activity with removal ability due to cell rupture. On the other hand, due to the reduction of the external discharge of surplus sludge, the phosphorus absorbed by phosphorus accumulating bacteria cannot be discharged from the system (Sarvajith and Nancharaiah, 2022). A reduction in the amount of sludge discharged in the reactor will prolong the SRT, and a longer SRT may also have a negative impact on phosphorus removal (de Sousa Rollemberg et al., 2022). The TN in the A2 device containing lysozyme was too high, which exceeded the system’s ability to accommodate the decomposition of nitrogen. The slight decrease in A3 and A4 was due to the hydrolysis of the enzyme preparation, which led to the sludge reduction and increased the sludge age of the activated sludge in the system. As a result, the denitrification ability of the system was enhanced. There was no significant difference in the NH_4_^+^-N removal rate between the experimental and control groups. During the experiment, the system had good nitrification capacity and stable operation, and the activity of ammonia-oxidizing bacteria in the sludge was good (Wang et al., 2020). The effluent concentration of NO_2_^−^-N did not change significantly, and the nitrite produced by ammonia oxidation could be oxidized to nitrate in time, so that there was no excessive accumulation of NO_2_^−^-N in the water. The NO_3_^−^-N content of A2 changed significantly, which seriously affected the denitrifying bacteria, and even died. The denitrification process was inhibited, A3 and A4 were reduced compared with A1, and the system reduced more nitrite to nitrogen gas from the water (Song et al., 2022).

### 3.3. Effects of enzyme preparation on microbial community structure

#### 3.3.1. Alpha diversity analysis

The diversity of the treated samples is shown in Table 2. A total of 10,945 OTUs were obtained in all the test samples, and the number of OTUs contained in A1-A4 ranged from 1,900 to 3,200. This indicates that the structure of the microbial community has changed. Alpha diversity statistics are shown in the Table 2. The sequencing coverage of all samples was more significant than 0.99, indicating that the sequencing results can reasonably reflect the microbial community structure in the samples. The number of OTUs was positively correlated with species richness. The number of OTUs of microbial communities in A2 and A3 was stable and increased compared with the control group. In contrast, the number of OTUs in A4 decreased significantly to 1921 due to the impact of neutral protease reagent, which destroyed the function of some microbial cells. The Chao1 index also showed a significant trend, indicating that the abundance of microbial communities in A2 and A3 was higher than that in A1, and the abundance of A4 was lower than that in A1. This may be due to the rapid occupation of niches by opportunistic populations in the presence of competing strains, and the subsequent dominant species eventually dominating community turnover and development, which leads to the separation of microbial community function and community stability over time scales (Wan et al., 2022). Compared with the A1 system, the Simpson index of A2, A3, and A4 fluctuated but not much, and had no response to the addition of enzyme agents. The Shannon indices of A1 and A2 were similar. The Shannon index of A3 was relatively increased, indicating an increase in community diversity. The Shannon index of A4 was decreased (Zhang et al., 2021). The Pielou_e index represents the evenness of the community, and its trend of the four reaction devices was the same as the previous analysis. Lysozyme and α-amylase had no significant effect on the microbial communities of species in the SBR system, and neutral protease caused a decrease in system diversity and abundance.

**Table 2:** Microbial community diversity indices of four SBRs.

Samples	OTUs	Chao1	Simpson	Shannon	Pielou_e	Goods_coverage
A1	2,802	2,837	0.972	7.884	0.689	0.994
A2	3,131	3,146	0.966	7.821	0.674	0.996
A3	3,091	3,106	0.989	8.752	0.755	0.996
A4	1921	1904	0.952	6.979	0.640	0.997

**Figure 2:**
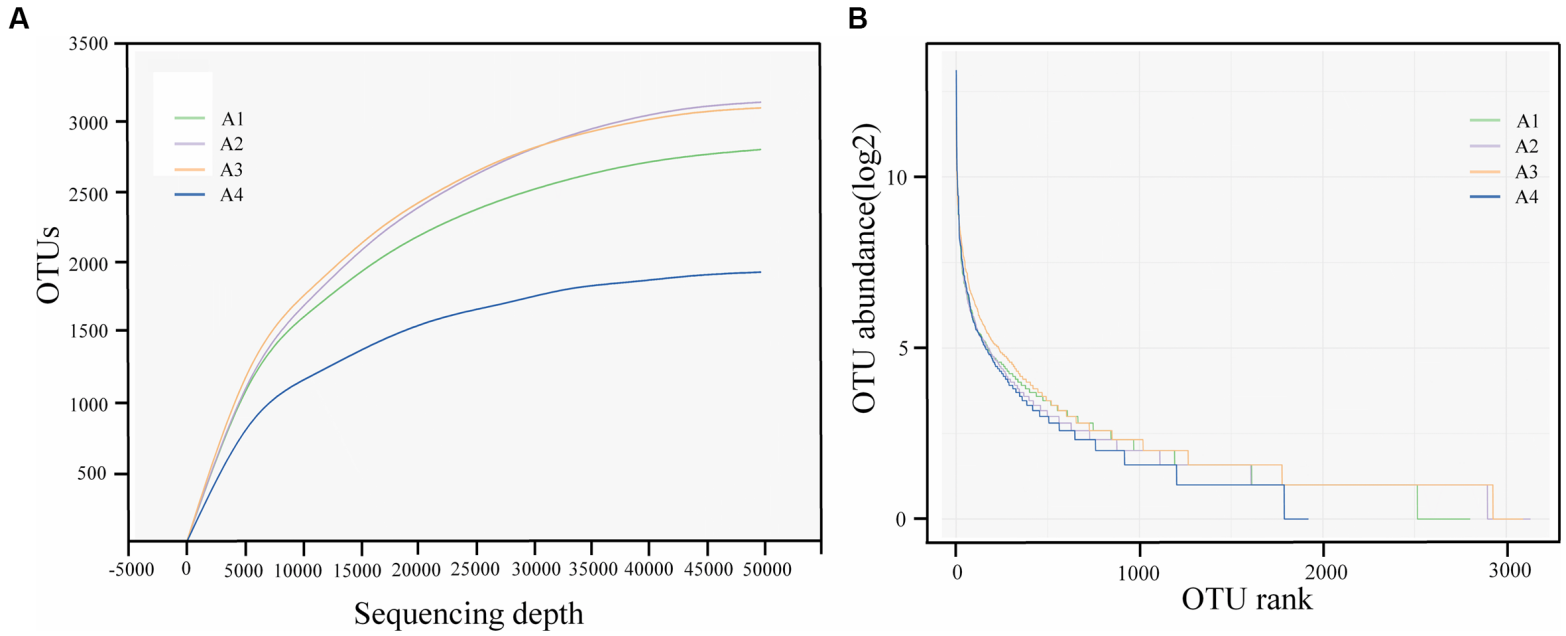
Alpha diversity analysis of samples A1-4: **(A)** Sparse curves; **(B)** Abundance rank curve (A1: Blank control A2: lysozyme A3: α-amylase A4: neutral protease).

Further analysis of the sparse curves in Figure 2A verified the information of valid reads and aggregated OTU in the three samples. At the same sequencing depth, the number of OTUs of the samples was in the following order: A2 > A3 > A1 > A4, which reflected the high diversity of the four samples and the difference in the effect of different enzyme reagents on the system. In addition, it can be seen from the sparse curve that the four samples in this experiment eventually reached a plateau, indicating that the amount of data for this sequencing was sufficient. The sequencing depth has basically covered all species in the sample, and the results can reflect the real situation of microbial diversity.

The abundance rank curve in Figure 2B shows the homogeneity of the microbial community based on the distribution of OTUs in each sample, which reflects the distribution pattern of abundance. In this curve, each OTU in the sludge was sorted according to the number of DNA sequences, and the abundance rank curves of OTU of A1, A2, A3, and A4 became relatively flat with the increase in the number of analyzed sequences, which indicated that the results of this study were reasonable and accurate. As shown in the figure, compared with A1, A2, and A3, A4 had a steeper fold, a greater different in community abundance, and lower bacterial diversity and homogeneity. In contrast, A2 and A3, with the addition of enzyme reagents, had smoother folds and contributed to the microbial diversity of the system.

#### 3.3.2. Beta diversity analysis

**Figure 3:**
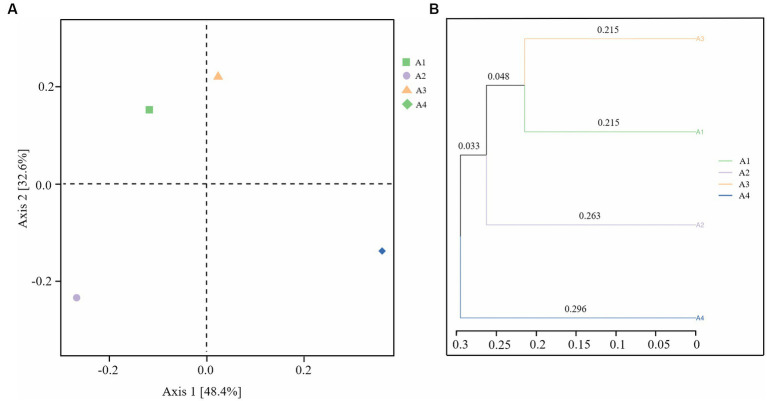
Beta diversity analysis of samples A1-4: **(A)** Principal coordinate analysis (PCoA); **(B)** UPGMA cluster analysis (A1: blank control A2: lysozyme A3: α-amylase A4: neutral protease).

Figure 3A shows the difference in the bacterial community among samples principal coordinate analysis (PCoA), in which the weighted UniFrac distance was calculated to more comprehensively reflect the similarities and differences between sample community compositions. From Figure 3A, principal coordinates 1 and 2 accounted for 48.4 and 32.6% of the total community variables, respectively. There were different groups in the four sludge samples. The results showed that there were deviations between the results of the experimental and control groups under different exposure conditions. The changes of microbial communities in A2 and A4 injected with lysozyme and neutral protease were much more obvious than those in A3, injected with α-amylase.

As shown in Figure 3B, UPGMA cluster analysis clustered the samples based on the similarity between them. The shorter the branch length between the samples, the more similar the two samples were. The results further showed that the neutral protease dosing treatment caused the greatest change in the microbial community, followed by lysozyme, while the microbial community in the device dosed with α-amylase was not significantly different from the control. These findings demonstrate that enzyme preparations can exert a suppressive effect on microorganisms while exerting a sludge reduction effect, significantly altering the composition of the microbial community.

#### 3.3.3. Species variation analysis and marker species

**Figure 4:**
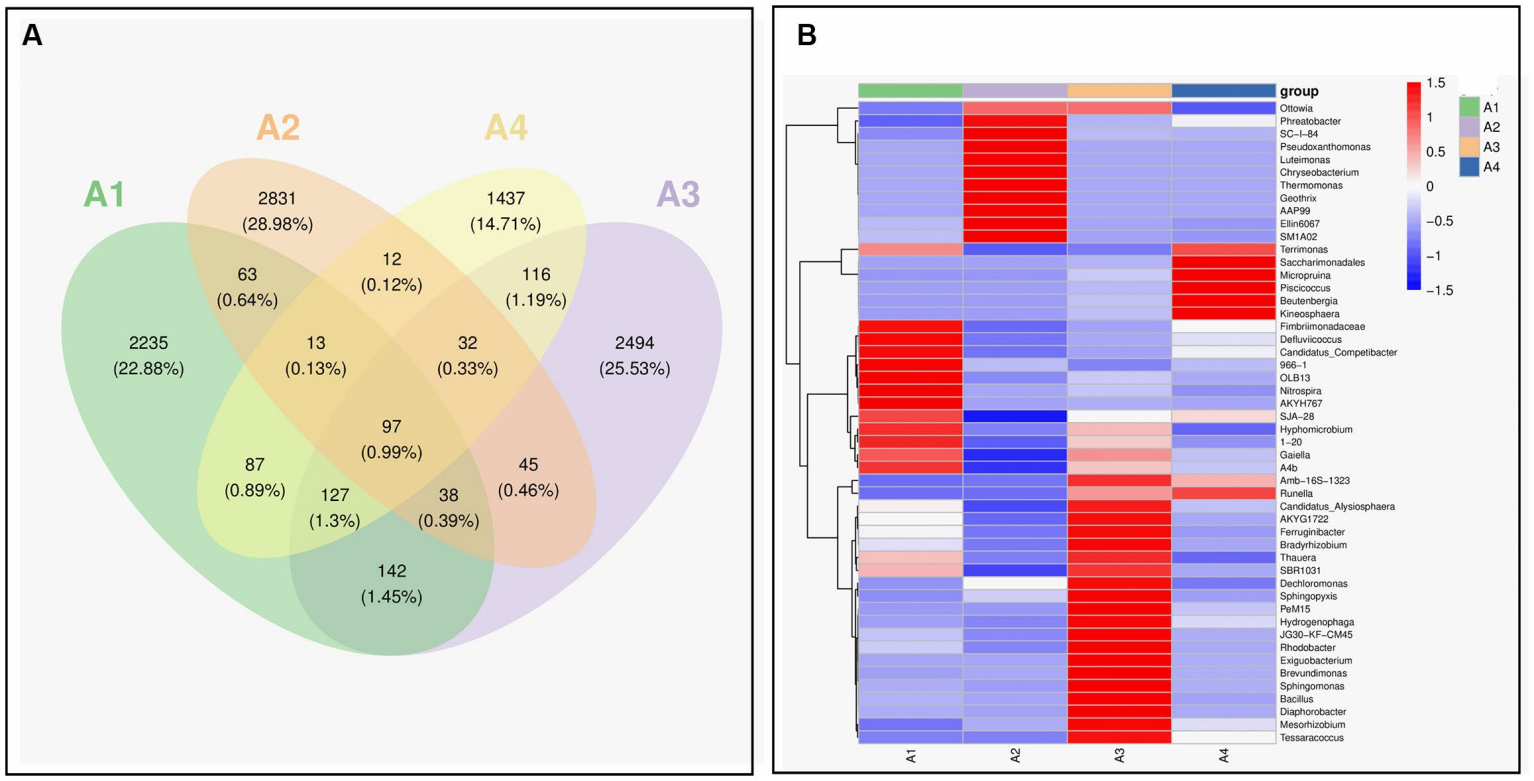
Species variation analysis and marker species of samples A1-4: **(A)** venn; **(B)** heat map (A1: blank control A2: lysozyme A3: α-amylase A4: neutral protease).

The Venn diagram in Figure 4A shows the changes in the microbial community structure in the four reaction systems. A total of 97 species were found in all samples, confirming the homology between A1, A2, A3, and A4. All four samples had a large number of different species, which was related to the dosing of different types of enzyme reagents, indicating a significant succession and change in bacterial community structure during the sludge initiation process. The abundance of microbial community in the system was increased, and the dominant flora was enriched, which was conducive to the stability of the system. The number of the same bacterial genus in the four types of sludge accounted for 3.5, 3.1, 3.1 and 5% of the total bacterial genera in A1, A2, A3 and A4, respectively, indicating that the species gradually increased during the operation process (Song et al., 2022).

The heat map in Figure 4B shows the trend of species abundance distribution of the samples, with red color blocks representing a relatively high abundance of the genus and blue color blocks representing a relatively low abundance of the genus. From the figure, all four samples are less closely related at the taxonomic level, with more pronounced differences in microbial composition and relative abundance. During evolution, only a few of the dominant genera in the control group ended up in slightly higher abundance in the A3 response group, while they were all at lower abundance levels in A2 and A4, with significant success in the microbial community.

### 3.4. Effects of microbial community structure

#### 3.4.1. Community structure analysis at the phylum level

**Figure 5:**
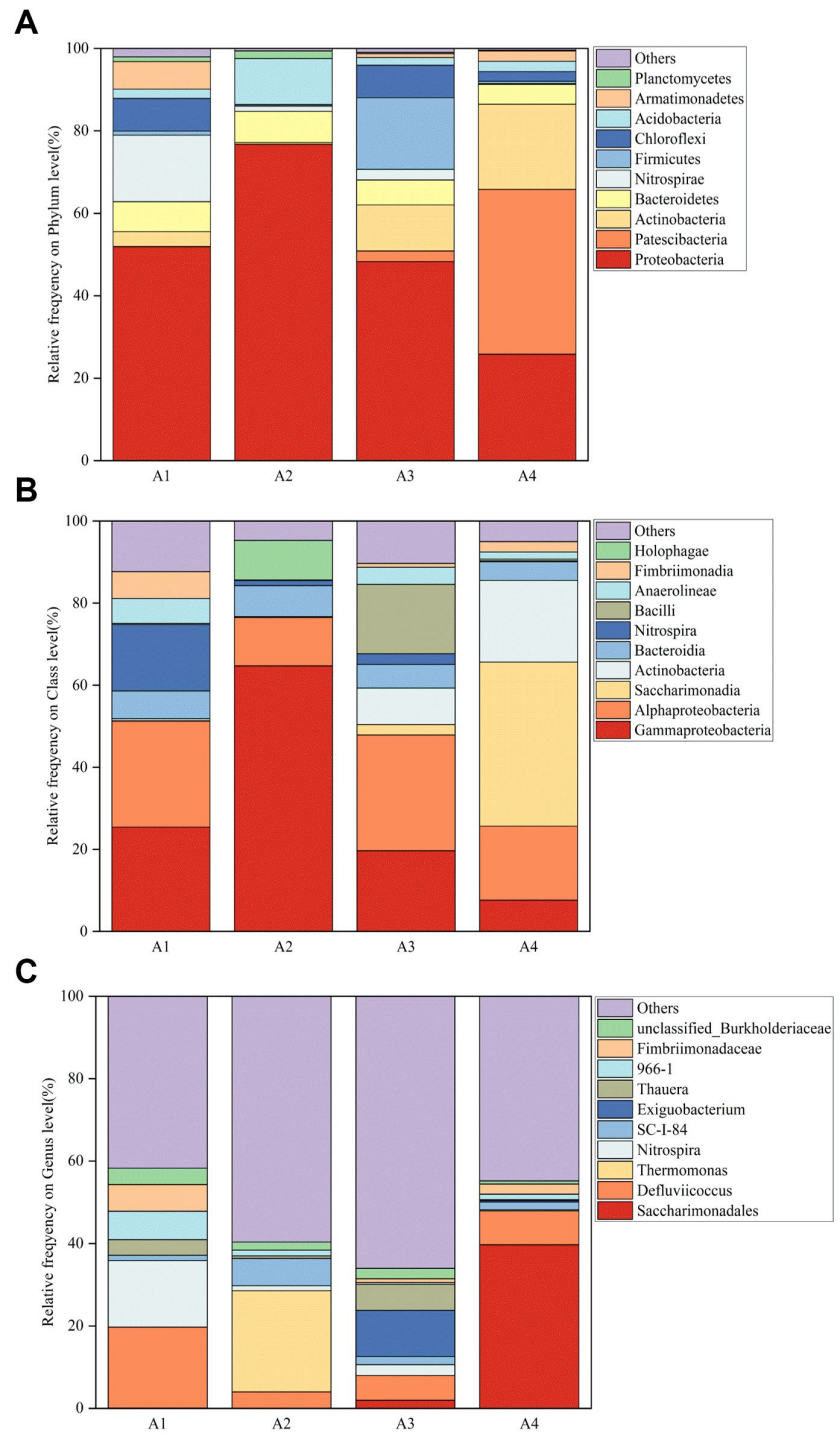
The microbial classification of bacterial 16S rDNA gene reads of samples A1-4: **(A)** phylum; **(B)** class; **(C)** genus (A1: Blank control A2: lysozyme A3: α-amylase A4: neutral protease).

In order to visualize the changing trend of microbial community structure in activated sludge, the species with the top 10 relative abundance of the microbial community at the phylum level in each activated sludge was selected, and the remaining species were combined and set as Others. As shown in Figure 5A. The dominant phylum included *Proteobacteria* (25.826–76.753%), *Patescibacteria* (0.087–39.991%), *Actinobacteria* (0.411–20.655%), *Bacteroidetes* (4.786–7.574%), *Nitrospirae* (1.210–17.319%). *Proteobacteria* are the largest phylum of bacteria. Most of the phosphorus-polymerizing bacteria belong to the Proteus. In addition, many bacteria can fix nitrogen and can effectively remove nitrogen and degrade organic matter (He et al., 2018), including AOB (Ammonia Oxidizing Bacteria), NOB (Nitrite Oxidizing Bacteria), and denitrifying bacteria, which are the main microorganisms involved in denitrification process and play an important role in anaerobic digestion (Su et al., 2020; Lu et al., 2021). The *Proteobacteria* of A2 increased to 76.8%, and the addition of lysozyme increased the bioavailable material content and dissolved sludge reflux, which promoted the growth of the *Proteobacteria* (Su et al., 2021). There was no significant difference in *Proteobacteria* of A3. The *Proteobacteria* of A4 decreased to as low as 25.8%, indicating a strong inhibition of the phosphorus removal effect of the system. Compared to A1, *Patescibacteria* increased by 99.8% in the reactor of A4. *Actinobacteria* are related to the settling performance of the system and can be involved in the degradation of refractory organic matter and the uptake of inorganic nitrogen and denitrification (Zhang et al., 2018). At the same time, it is also a common category of phosphorus removal bacteria, including a variety of metabolic species, with the function of nitrogen and phosphorus removal. *Actinobacteria* of A2 showed a decreasing trend, corresponding to a sharp increase in its TN and NO_3_^−^-N content. *Actinobacteria* of A3 and A4 showed an increasing trend, probably due to their adaptation and resistance to enzymatic agents. *Bacteroidetes* are common bacteria in wastewater treatment, usually participating in wastewater treatment as heterotrophic bacteria and denitrification functional bacteria (Pan et al., 2020), and playing an important role in COD degradation and denitrification and phosphorus removal (Yao et al., 2021). It can decompose proteins, carbohydrates and other macromolecules, and nitrify to remove nitrogen (Seo et al., 2019), effectively degrade organic pollutants under anaerobic conditions. *Bacteroidetes* and *Proteobacteria* are considered a common functional phylum in wastewater treatment systems. Compared to A1, *Bacteroidetes* of A2 were mostly unchanged, and *Bacteroidetes* of A3 and A4 were decreasing. *Nitrospirae* is mainly responsible for nitrification processes, including autotrophic metabolism and subsequent nitrite oxidation (Luo et al., 2013). *Nitrospirae* in A2, A3, and A4 all decreased sharply, affecting the nitrification–denitrification process of the system. Compared to A1, the number of phyla in A3 and A4 increased significantly and the number of phyla in A2 decreased significantly, indicating that the action of enzyme preparation on SBR system had a great influence on the microbial community structure.

#### 3.4.2. Community structure analysis at the class level

The species with the top 10 relative abundance of microbial communities at the class level in each activated sludge were selected, and the remaining species were combined and set as Others. As shown in Figure 5B, the dominant bacterial class in the A1 blank control group included *Gammaproteobacteria* (25.429%), *Alphaproteobacteria* (25.822%), *Bacteroidia* (6.738%), *Nitrospira* (16.157%), and *Fimbriimonadia* (6.514%). All of them contributed to the nitrification–denitrification performance in the system and the reactor operated well. The top three bacteria in relative abundance play an important role in degrading nitrogen-containing compounds (Liu Q. et al., 2022; Liu X. et al., 2022; Liu Z. et al., 2022). Due to the addition of lysozyme in A2, the dominant class during the evolution became *Gammaproteobacteria* (64.714%), *Alphaproteobacteria* (11.771%), *Bacteroidia* (7.553%), *Holophagae* (9.631%), and *Nitrospira* (1.21%). The reagent dosing promoted the growth of some dominant bacteria, exemplified by the increase in the relative abundance of *Holophagae*, a strictly anaerobic bacterium belonging to the *Acidobacteria* phylum. The similarity in microbial composition between reactors A3 and A1 is related to the domestication process of the reactors in the system. The dominant classes in A4 were *Gammaproteobacteria* (7.647%), *Alphaproteobacteria* (18.031%), *Saccharimonadia* (39.983%), *Actinobacteria* (19.868%), and *Bacteroidia* (4.516%). While the sludge was reduced, the effluent water quality was good due to the removal of nitrogen and phosphorus and the removal of organic matter by the dominant bacteria. It can be seen that the relative abundance of *Bacteroidia* in the four reactors is at a relatively stable level, and the slight differences may be due to the death of other classes. In general, the microorganisms that are not adapted to the system will gradually be eliminated with the operation of the reactor, and finally show the difference of microbial community structure (He et al., 2022).

#### 3.4.3. Community structure analysis at the genus level

The species with the top 10 relative abundance of microbial communities at the genus level in each activated sludge were selected, and the remaining species were combined and set as Others. As shown in Figure 5C, A1 dominant genera were *Defluviicoccus* (19.624%), *Nitrospira* (6.157%), *966–1* (6.916%), *Fimbriimonadaceae* (6.502%), and *unclassified_Burkholderiaceae* (3.942%). *Defluviicoccus* belongs to a genus of Gram-negative cocci that is usually highly abundant in wastewater treatment plants and can be used for phosphorus removal (Maszenan et al., 2022). *Nitrospira* is an aerobic autotrophic bacterium, which is considered to be the most common and abundant NOB in sewage treatment (Martins et al., 2020), and can oxidize nitrite into nitrate. In the reactor with lysozyme, α-amylase and neutral protease added, the dominant bacteria evolved. On the one hand, due to the impact of reagents on the system, the relative abundance changed; on the other hand, due to the reduction of sludge discharge, the system would lead to a certain degree of sludge aging, and microorganisms would metabolize to alleviate the aging of the sludge (Liu Q. et al., 2022; Liu X. et al., 2022; Liu Z. et al., 2022). *Thermomonas* is a denitrifying genus in wastewater treatment systems, which is beneficial to denitrification. *Thermomonas* reached a relative abundance of 24.566% in A2. The relative abundance of *Exiguobacterium* (11.238%) and *Thauera* (6.302%) were higher in A3. *Exiguobacterium* is a Gram-positive species, which acts in nutrient fixation and degradation of toxic substances. *Thauera* belongs to denitrifying bacteria, which is an important function in the denitrification and degradation of pollutants in the system, and also has the function of EPS secretion. *Saccharimonadales* (39.692%) and *Defluviicoccus* (8.259%) were more abundant in A4, *Saccharimonadales* survive in an anaerobic environment and participate in denitrification (Huang et al., 2022). *Defluviicoccus* has a phosphorus removal effect.

## 4. Conclusion

This study revealed that using enzymes to act on the SBR system can promote sludge hydrolysis and achieve volume reduction. By comparing the accumulated excess sludge emissions during the experimental process, it can be shown that after treatment with different enzyme preparations, different degrees of effect were obtained, among which, lysozyme had the best sludge hydrolysis effect of 48.5%, followed by neutral protease (31%) and α-amylase (22.5%).

The four sludge samples were sequenced with high throughput, and the richness and diversity of the sludge community supplemented with lysozyme and α-amylase were higher. Compared with the control group, the sludge community supplemented with lysozyme and protease was significantly different, and the community succession was obvious. At the three levels of phylum, class and genus, the dominant species of bacteria in different reactors did not change much, but their abundance was different, and the content of bacteria related to water treatment was reduced, which affected the operation ability of the system. The feasibility and defects of enzyme treatment for sludge reduction were proved. Next, the operation time should continue to be extended, and in the case of complex actual wastewater, the experimental details should be further optimized to improve the shortcomings of the treatment process, so that it can be run in actual wastewater treatment applications.

## Data availability statement

The original contributions presented in the study are included in the article/Supplementary material, further inquiries can be directed to the corresponding author.

## Author contributions

JW: conceptualization, investigation, and writing-original draft. SL: investigation, methodology, and resources. ZH and MJ: investigation and visualization. All authors contributed to the article and approved the submitted version.

## Funding

We appreciate the financial support from Scientific Research Foundation of Anhui Universities (KJ2021A0442), Start-up Foundation for High-level Introduction Talents of Anhui University of Science and Technology (2021yjrc04) and State Key Laboratory of Pollution Control and Resource Reuse Foundation (No. PCRRF21040).

## Conflict of interest

The authors declare that the research was conducted in the absence of any commercial or financial relationships that could be construed as a potential conflict of interest.

## Publisher’s note

All claims expressed in this article are solely those of the authors and do not necessarily represent those of their affiliated organizations, or those of the publisher, the editors and the reviewers. Any product that may be evaluated in this article, or claim that may be made by its manufacturer, is not guaranteed or endorsed by the publisher.
