# Supplementary material for: Changes in microbial community during hydrolyzed sludge reduction
Source: Front Microbiol. 2023 Sep 1;14:1239218. doi: 10.3389/fmicb.2023.1239218 (PMC10502510; doi:10.3389/fmicb.2023.1239218)
Supplement: Supplementary file 1 [file Data_Sheet_1.docx]

**Changes in Microbial Community during Hydrolyzed Sludge Reduction**

Jiating Wu^1,2^, Shaomin Liu^1,2^*, Ziyan Hu^1,2^, Mengyu Jiang^1,2^, Jinglin Zhu^1,2^

^1.^ School of Earth and Environment, Anhui University of Science and Technology, Huainan 232001, China

^2.^ State Key Laboratory of Mining Response and Disaster Prevention and Control in Deep Coal Mines (Anhui University of Science and Technology), Huainan 232001, China

*To whom correspondence should be addressed.

E-mail: [shmliu1@163.com](mailto:shmliu1@163.com)

**Supplementary data**

**Fig.S1.** Effects of different enzyme preparations on SVI

**Fig.S2.** Effects of effluent quality: (a) COD; (b) TP concentrations

**Fig.S3.** Effects of the nitrification and denitrification capacity: content of NH_4_^+^-N, NO_2_^-^-N, and NO_3_^-^-N

**Fig.S4.** Effects of reaction conditions on sludge surface morphology: (a) Control group; (b)lysozyme; (c) α-amylase; (d) neutral protease





**Fig.S1.** Effects of different enzyme preparations on SVI





**Fig.S2.** Effects of effluent quality: (a) COD; (b) TP concentrations





**Fig.S3.** Effects of the nitrification and denitrification capacity: content of NH_4_^+^-N, NO_2_^-^-N, and NO_3_^-^-N





**Fig.S4.** Effects of reaction conditions on sludge surface morphology: (a) Control group; (b)lysozyme; (c) α-amylase; (d) neutral protease
